# Genetic background in late-onset sensorineural hearing loss patients

**DOI:** 10.1038/s10038-021-00990-2

**Published:** 2021-11-26

**Authors:** Natsumi Uehara, Takeshi Fujita, Daisuke Yamashita, Jun Yokoi, Sayaka Katsunuma, Akinobu Kakigi, Shin-ya Nishio, Ken-ichi Nibu, Shin-ichi Usami

**Affiliations:** 1 Department of Otolaryngology-Head and Neck Surgery, Kobe University Graduate School of Medicine, Kobe, Japan; 2 Department of Hearing Implant Sciences, Shinshu University School of Medicine, Matsumoto, Japan

**Keywords:** Genetic testing, Genetics research

## Abstract

Genetic testing for congenital or early-onset hearing loss patients has become a common diagnostic option in many countries. On the other hand, there are few late-onset hearing loss patients receiving genetic testing, as late-onset hearing loss is believed to be a complex disorder and the diagnostic rate for genetic testing in late-onset patients is lower than that for the congenital cases. To date, the etiology of late-onset hearing loss is largely unknown. In the present study, we recruited 48 unrelated Japanese patients with late-onset bilateral sensorineural hearing loss, and performed genetic analysis of 63 known deafness gene using massively parallel DNA sequencing. As a result, we identified 25 possibly causative variants in 29 patients (60.4%). The present results clearly indicated that various genes are involved in late-onset hearing loss and a significant portion of cases of late-onset hearing loss is due to genetic causes. In addition, we identified two interesting cases for whom we could expand the phenotypic description. One case with a novel *MYO7A* variant showed a milder phenotype with progressive hearing loss and late-onset retinitis pigmentosa. The other case presented with Stickler syndrome with a mild phenotype caused by a homozygous frameshift *COL9A3* variant. In conclusion, comprehensive genetic testing for late-onset hearing loss patients is necessary to obtain accurate diagnosis and to provide more appropriate treatment for these patients.

## Introduction

Sensorineural hearing loss (SNHL) is one of the most common sensory disorders in humans [1, 2]. The incidence of the congenital SNHL is estimated to be 1–2 in 1000 newborns, among which at least 60% are presumed to be associated with genetic causes [3]. Sloan-Heggen et al. undertook the genetic analysis of congenital deafness patients by targeted genomic enrichment with massively parallel DNA sequencing (TGE + MPS) and identified the causative gene mutations in 44% of cases [3]. In Japan, Mori et al. reported that the diagnostic rate was 41% in congenital or early-onset (<6 year) hearing loss (HL) patients based on screening for 154 mutations in 19 deafness genes using MPS combined with Invader assay and TaqMan genotyping [4]. On the other hand, these studies also reported that the diagnostic rates in late-onset hearing loss patients were lower than those in early-onset patients (28% and 16%, respectively) [3, 4]. Late-onset SNHL is believed to be a complex disorder, associated with age-related hearing loss, idiopathic sudden SNHL, acoustic neuroma, chronic otitis media or environmental risk factors (including noise exposure and ototoxic drug exposure). However, a certain number of late-onset bilateral symmetrical HL cases are thought to involve genetic factors, particularly in those with progressive HL presenting as worse than the average hearing for age. At present, a majority of late-onset SNHL cases do not receive genetic testing; thus, the etiology of late-onset SNHL remains largely unknown.

In this study we focused on late-onset bilateral SNHL patients and aimed to show the frequency of hereditary HL as well as describe the clinical features of these cases.

## Materials and methods

This study was conducted with the approval of the Ethics Committee of Kobe University Graduate School of Medicine (Approval number:170081). Written informed consent was obtained from all subjects. All procedures were performed in accordance with the Guidelines for Genetic Tests and Diagnoses in Medical Practice of the Japanese Association of Medical Sciences and the tenets of the Declaration of Helsinki.

### Subjects

Sixty-four unrelated patients with bilateral SNHL were enrolled in this study. We defined late-onset HL as the HL with an age at onset of 6 years of age and over and, based on this definition, we excluded cases with congenital or pre-lingual onset SNHL (with an age at onset of under 6 years of age). In addition, we also excluded patients aged over 60 years at SNHL onset to remove cases of presbycusis. Finally, 48 unrelated Japanese patients with late-onset bilateral SNHL who underwent clinical genetic testing between April 2012 and April 2020 at Kobe University Graduate School of Medicine participated in this study (Table 1).

**Table 1:** Patients characteristics

Characteristic	Number	%
**Number of subjects**	48	100.0
**Sex**		
Female	25	52.1
Male	23	47.9
**Family history of HL** (+)	22	45.8
(−)	26	54.2
**Age at onset**		
6–10 y.o.	9	18.8
11–20 y.o.	4	8.3
>20 y.o.	35	72.9
**Severity**		
mild/mild	5	10.4
moderate/moderate	19	39.6
severe/severe	4	8.3
profound/profound	4	8.3
normal/mild	1	2.1
mild/moderate	3	6.3
moderate/severe	7	14.6
moderate/profound	3	6.3
profound/severe	2	4.2
**Audiometric configuration**		
Flat	15	31.3
Gently sloping	13	27.1
Steeply sloping	10	20.8
Deaf	3	6.3
U-shaped	3	6.3
Different types on each side	4	8.3

*y.o.* years old, *HL* hearing loss.

### Clinical evaluations

Hearing thresholds were evaluated using pure-tone audiometry (PTA) and classified by pure-tone average over 500, 1000, 2000, and 4000 Hz. The severity of HL was classified into mild (21–40 dB HL), moderate (41–70 dB HL), severe (71–95 dB HL), and profound (>95 dB HL). The audiometric configurations were categorized into low-frequency, mid-frequency (U-shaped), high-frequency (gently sloping type and steeply sloping type), flat type, and deaf, as reported previously [5]. The data for age at onset of HL, the progressiveness of HL and family history were obtained from medical charts.

### Amplicon resequencing and variant annotation

Amplicon libraries were prepared using an Ion AmpliSeq™ Custom Panel for 68 genes reported to cause non-syndromic hereditary HL (ThermoFisher Scientific, MA, USA), in accordance with the manufacturer’s instructions. The detailed protocol has been described elsewhere [6]. MPS was performed with an Ion Proton system using an Ion HiQ Chef Kit and an Ion P1 Chip (ThermoFisher Scientific). The sequence data were mapped against the human genome sequence (build GRCh37/hg19) with a Torrent Mapping Alignment Program. After sequence mapping, the DNA variant regions were piled up with Torrent Variant Caller plug-in software. After variant detection, their effects were analyzed using ANNOVAR software [7, 8]. The missense, nonsense, insertion/deletion and splicing variants were selected from among the identified variants. Variants were further selected as less than 1% of: (1) the 1000 genome database, (2) 6500 exome variants, (3) the Human Genetic Variation Database (a dataset for 1208 Japanese exome variants), and (4) 333 in-house Japanese normal hearing controls. This filtering process was performed using our original database software described elsewhere [9]. The pathogenicity of selected variants was evaluated by American College of Medical Genetics (ACMG) standards and guidelines [10]. For missense variants, in particular, functional prediction software, including Sorting Intolerant from Tolerant (SIFT), Polymorphism Phenotyping (PolyPhen2), LRT, Mutation Taster, Mutation Assessor, REVEL, and CADD, were used through the ANNOVAR software program [7, 8]. Direct sequencing was utilized to confirm the selected variants. Copy number variation (CNV) analysis was performed by using the read depth data with our published copy number variation detection method for Ion AmpliSeq enrichment and Ion PGM/Proton/S5 sequencing as described previously [11]. All genetic analyses were performed in Shinshu University School of Medicine as a collaborative study.

## Results

### Patient characteristics and identified variants

The age at onset of participants ranged from 6 to 60 years. Among them, 9 cases experienced onset in their 1st decade (6–10 years old) and the other 42 cases experienced onset in their 2nd decade or later (11–60 years old), accounting for 81.2% of all participants (Table 1).

As for the audiometric configuration, flat-type HL was the most common, being observed in 15 cases (31.3%), followed by 13 cases with gently sloping-type HL (27.1%), 10 cases with steeply sloping-type HL (20.8%), three cases with profound-type HL (6.3%), three cases with U-shaped-type HL (6.3%), and four cases with different types of HL in the left and right ears (Table 1).

We identified 25 possibly disease-causing variants from 29 probands, affording a diagnostic rate for this study of 60.4%. The most prevalent causative gene for late-onset HL in this study was a mitochondrial m.3243A>G mutation, which was observed in 6 cases, followed by four cases with *COCH* gene variants, three cases each with *CDH23*, *KCNQ4* and *MYO6* variants, two cases with *EYA4* variants, and one case each with *ACTG1*, *COL9A3*, *GJB2*, *MYO7A*, *POU4F3*, *STRC*, *USH2A*, and mitochondria m.1555A>G variants (Table 2). Among the 25 identified variants, 18 had been reported previously as causative variants, and 7 were novel variants (Table 2). The novel variants consisted of two missense variants, one nonsense variant, three frameshift variants and one splicing variant. Based on the ACMG guidelines, the novel variants were categorized as pathogenic (1), likely pathogenic (7) and variants of uncertain significance variant (1) (Table 3).

**Table 2:** Summary of the causative variants identified in this study

Gene	Nucleotide change	Amino acid change	Onset age	Age	Inheritance	Gender	Severity of HL (L/R)	Audiometric configuration	Reference
*MT-TL1*	m.3243A>G		30	79	Sporadic	M	profound/severe	Flat/Profound	van den Ouweland (1992) [36]
*MT-TL1*	m.3243A>G		30	55	Maternal	M	profound/moderate	Profound/Flat	
*MT-TL1*	m.3243A>G		20	45	Maternal	F	moderate/moderate	Flat	
*MT-TL1*	m.3243A>G		40	60	Maternal	M	severe/moderate	Flat	
*MT-TL1*	m.3243A>G		20’s	43	Maternal	M	moderate/moderate	Flat	
*MT-TL1*	m.3243A>G		20’s	34	Maternal	M	severe/severe	Flat	
*COCH*	c.1115T>C	p. I372T	40	53	AD	F	severe/moderate	Steeply sloping	Tsukada (2015) [27]
*COCH*	c.1115T>C	p. I372T	50	53	AD	M	severe/moderate	Gently sloping	Tsukada (2015) [27]
*COCH*	c.1115T>C	p. I372T	10	36	AD	F	mild/mild	Steeply sloping	Tsukada (2015) [27]
*COCH*	c.236A>G	p.H79R	20’s	43	Sporadic	M	moderate/moderate	Gently sloping	This study
*CDH23*	c.719C>T/c.4762C>T	p.P240L/R1588W	40	59	AR	F	moderate/moderate	Steeply sloping	Wagatsuma (2007) [37]
*CDH23*	c.719C>T/c.4762C>T	p.P240L/R1588W	50	52	AR	F	severe/moderate	Steeply sloping	Wagatsuma (2007) [37]
*CDH23*	c.4762C>T homo	p.R1588W	50	58	AR	F	severe/severe	Steeply sloping	Miyagawa (2013) [6]
*MYO6*	c.3496C>T	p.R1166X	12	36	Sporadic	M	severe/severe	U-shaped	Ahmed (2003) [38]
*MYO6*	c.1015C>T	p. R339W	40	57	AD	M	moderate/moderate	Gently sloping	Yang (2013) [39]
*MYO6*	c.2393G>A	p. W798X	20’s	33	AD	F	mild/moderate	U-shaped	This study
*KCNQ4*	c.211delC	p. Q71fs	10’s	30	AD	F	moderate/moderate	Steeply sloping	Kamada (2006) [40]
*KCNQ4*	c.1656dupA	p. L553Tfs*11	35	37	AD	F	moderate/moderate	Flat	This study
*KCNQ4*	c.961G>A	p. G321S	20	36	Sporadic	M	moderate/moderate	Flat	Coucke (1999) [41]
*EYA4*	c.1777delG	p. G593Afs*4	40	52	AD	F	severe/severe	Flat	This study
*EYA4*	c.1886_1899del	p. A629fs	40	54	AD	M	moderate/moderate	Gently sloping	Shinagawa (2020) [23]
*GJB2*	c.176191del/c.235delC	p. G59fs/p. L79fs	10’s	51	AR	F	moderate/moderate	Gently sloping	Abe (2000) [42] /Fuse (1999) [43]
*MT-RNR*	m.1555A>G		6	58	Sporadic	M	profound/profound	Steeply sloping	Prezant (1993) [44]
*MYO7A*	c.1667GT/c.1369G>A	p.G556V/p. A457T	8	47	AR	M	profound/profound	Profound	Bakon (2016) [13]/This study
*COL9A3*	c.1587dupT homo	p.G530Wfs*71	20’s	58	AR	F	severe/moderate	Flat	This study
*ACTG1*	c.833C>T	p.T278I	30	59	AD	F	profound/profound	Profound	van Wijk E (2003) [22]
*POU4F3*	c.976A>T	p.R326X	10’s	40	AD	M	moderate/moderate	Gently sloping	Kitano (2017) [45]
*STRC*	2 copy loss		20’s	21	AR	F	mild/mild	Gently sloping	Yokota (2019) [46]
*USH2A*	c.8559-2A>G/c.6806-2A>C		30	61	AR	F	moderate/profound	Gently sloping	Dai H (2008) [47]/This study

*HL* hearing loss.

**Table 3:** In silico prediction scores and pathogenicity classification of novel variants identified in this study

Case No.	Gene	Inheritance	Nucleotide change	Amino acid change	SIFT	PP2	LRT	MutT	MutA	ACMG
Case 1	*COCH*	AD	c.236A>G	p.H79R	D	D	D	D	H	Uncertain significance (PM2, PP3)
Case 2	*USH2A*	AR	c.8559-2A>G		–	–	–	D	–	Previously reported
			c.6806-2A>C		–	–	–	D	–	Pathogenic (PVS1, PM2, PP1)
Case 3	*KCNQ4*	AD	c.1656dupA	p.L553Tfs*11	–	–	–	–	–	Likely pathogenic (PVS1, PM2)
Case 4	*EYA4*	AD	c.1777delG	p.G593Afs*4	–	–	–	–	–	Likely pathogenic (PVS1, PM2)
Case 5	*MYO6*	AD	c.2393G>A	p.W798X	–	–	D	A	–	Likely pathogenic (PVS1, PM2)
Case 6	*MYO7A*	AR	c.1369G>A	p.A457T	D	P	D	D	L	Likely pathogenic (PM2, PM3, PM5, PP3)
			c.1667G>T	p.G556V	D	D	D	D	H	Previously reported
Case 7	*COL9A3*	AR	c.1587dupT (homo)	p.G530Wfs*71	–	–	–	–	–	Likely pathogenic (PVS1, PM2)

*PP2* Polyphen-2, *Mut T* Mutation Taster, *MutA* Mutation Assessor, *D* damaging or deleterious, *T* tolerated, *P* possibly or probably damaging, *A* disease-causing automatic, *L* low, *M* medium, *H* high.

### Clinical features of patients with novel variants

**Fig. 1:**
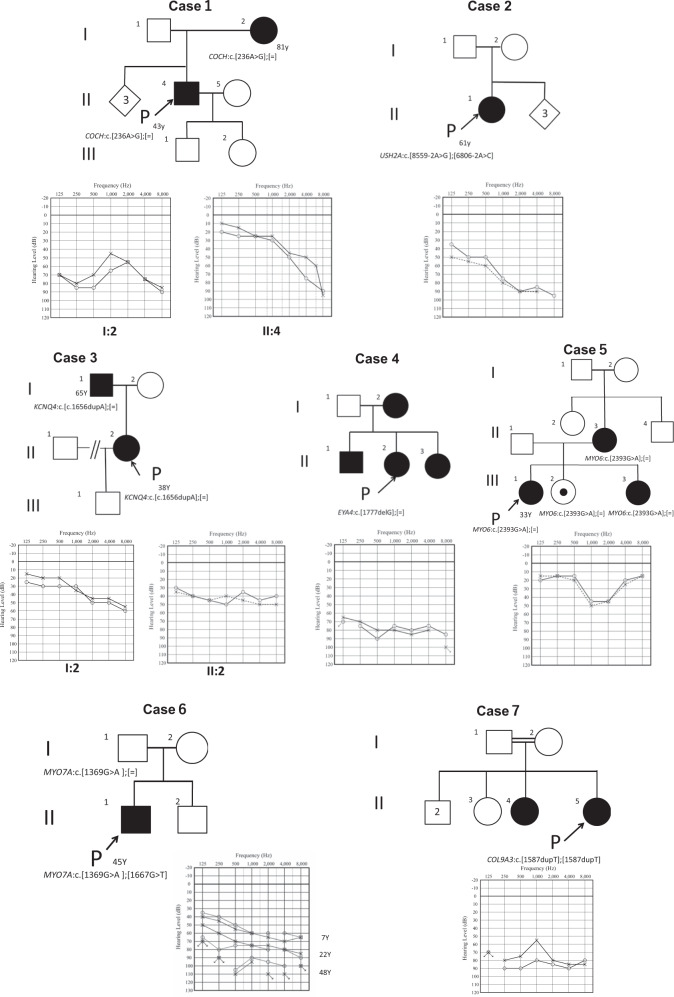
Pedigrees and audiograms of the seven families who carried a possible pathogenic variant identified in this study. Filled symbols indicate affected individuals. Arrows indicate probands in each family. Case 6 also showed progression of hearing loss from 7 years of age

Table 3 and Fig. 1 summarize the clinical features of the 7 individuals with novel variants.

Case 1 is a 43-year-old male. At around the age of 20, he experienced repeated episodes of dizziness and his HL gradually progressed. His mother had also experienced repeated episodes of dizziness with HL when she was in her twenties. Genetic analysis of this patient identified a heterozygous *COCH* gene missense variant (*COCH*: NM_004086:c.236A>G:p.H79R). His mother was found to carry the same variant.

Case 2 is a 61-year-old female who became aware of a loss of vision and HL in her thirties. She was diagnosed with retinitis pigmentosa through ophthalmological testing. Her HL gradually progressed and she was clinically suspected of Usher syndrome. She had never experienced any episodes of vertigo. Genetic analysis of this patient identified compound heterozygous *USH2A* gene splicing variants. *USH2A*: NM_206933:c.8559-2A>G was previously reported as a pathogenic variant of Usher syndrome type 2, and the other is novel splicing variant (NM_206933:exon36:c.6806-2A>C).

Case 3 is a 37-year-old female. She visited an otolaryngologist with a chief complaint of tinnitus and was diagnosed with HL when she was aged 35. Her audiometric configuration was of the gently sloping type at first, but HL progressed gradually even in the lower frequencies. She complained of dizziness about once every two years. Her father also had the same type of HL. Genetic analysis of this patient identified a heterozygous *KCNQ4* frameshift variant (*KCNQ4*: NM_004700:c.1656dupA:p.L553Tfs*11).

Case 4 is a 52 -year-old female, with HL diagnosed during a checkup at the age of 40. She started to use hearing aids at the age of 50. Her mother, brother and sister had also experienced HL from their forties, and they also used hearing aids. Genetic analysis of this patient identified a heterozygous *EYA4* gene frameshift variant (*EYA4*: NM_004100:c.1777delG:p.G593Afs*4).

Case 5 is a 33-year-old female, with a history of HL from the age of 27. Her HL progressed with age. Her mother had suffered HL from her forties. Her youngest sister had also suffered HL from elementary school. Genetic analysis of this patient identified a heterozygous *MYO6* gene nonsense variant (*MYO6*: NM_004999: c.2393G>A:p.W798X). Her mother and sisters also carried the same variant. Her middle sister is presumed not to have developed HL yet.

Case 6 is a 47 -year-old male, diagnosed with HL during a checkup at age 7, who started wearing hearing aids when he was 10 years old. His HL was considered to be gradually progressive. He had no episodes of vertigo. The genetic testing revealed compound heterozygous variants in the *MYO7A* gene. *MYO7A*: NM_000260:c.1667G>T:p.G556V was previously reported [12] as a causative variant for retinitis pigmentosa and other is the novel variant *MYO7A*: NM_000260:c.1369G>A:p.A457T. His father had normal hearing and had only the c.1369G>A variant, thus these two variants are in the *trans* allele. We therefore recommended him to visit the department of ophthalmology, and he was diagnosed with retinitis pigmentosa. He received a cochlear implant (CI) in the left ear at the age of 48 to benefit future communication. CI was effective for this case and his ability to understand speech was improved.

Case 7 is a 58-year-old female, diagnosed with HL in her twenties. Her parents were first cousins and her elder sister also had HL. Genetic analysis of this patient identified a homozygous *COL9A3* gene frame-shift variant (*COL9A3*: NM_001853:c.1587dupT:p.G530Wfs*71).

This patient did not have any arthritis, but had cataracts and retinal detachment, which are known to be associated with Stickler syndrome.

## Discussion

In this study, we analyzed 48 late-onset SNHL patients, and identified 25 possibly disease-causing variants in 29 cases (60.4%). Among the 25 identified variants, 7 were novel. Genetic testing for HL, particularly in congenital or early-onset cases, is now widely available due to its clinical benefits in providing accurate diagnosis, prediction of HL severity, estimation of associated symptoms, selection of appropriate habilitation options, prevention of HL, and better genetic counseling [13]. In this study we confirmed the usefulness of genetic testing for late-onset HL cases, even though it is not commonly performed at present.

The diagnostic rate (60.4%) in this study was higher than those of previous studies (28% and 16%) [3, 4]. As our institution is a university hospital (tertiary referral hospital), and many cases in this study have multiple affected family members, it might explain the increased diagnostic ratio of this cohort. Indeed, 45.8 % (22/48) of our cohort have affected family members. In a previous study, the diagnostic rate in sporadic cases was 19%, which was lower than that of autosomal recessive or autosomal dominant cases (35% and 35%, respectively) [14]. In addition, our cohort also included the patients with a maternal family history of suspected mitochondrial disorders, which may also have increased the diagnostic rate. Mitochondrial mutations (m.1555A>G or m.3243A>G) were identified in 15% (7/48) of patients in this study. As another cause of the higher diagnostic rate, our cohort included many cases with progressive HL. In a previous study, the diagnostic rate for progressive HL cases was higher than that for stable HL cases [14]. These factors may have led to the higher diagnostic rate in this study. From a diagnostic perspective, these clinical characteristics will be useful for the selection of candidates for genetic testing to increase the diagnostic yield and efficiency.

A previous paper showed that the responsible genes differ between congenital or early-onset HL and late-onset HL [4]. In this study, we also identified many causative genes which was reported as genetic causes of late-onset and/or progressive HL, such as the *KCNQ4*, *COCH*, *CDH23*, *EYA4*, *MYO7A*, *MYO6, ACTG1* and mitochondrial genes. As HL due to these genes is more or less progressive, the most appropriate therapeutic option from among hearing aids, electric acoustic stimulation (EAS), and cochlear implantation (CI) needs to be considered. Etiology as well as rate of progression is important to decision making, and genetic testing can provide this information [15]. If the etiology is located within the cochlea, good performance can be expected after CI [15].

The *CDH23* gene is known to cause either Usher syndrome type 1D (USH1D) or non-syndromic HL (DFNB12). The phenotype range of *CDH23*-associated HL varies from congenital profound HL to adult-onset high-frequency-involved HL [16]. CI has been applied for patients with insufficient amplification by hearing aids, and EAS devices are a good therapeutic option for patients with residual hearing [15, 17, 18]. In this study, two of three DFNB12 cases who did not have sufficient amplification by hearing aids received CI and showed good performance.

The *MYO7A* gene is known to cause autosomal dominant or autosomal recessive non‐syndromic HL (DFNA11/DFNB2) as well as Usher syndrome (USH1B), [19, 20] which is characterized by congenital, bilateral, profound sensorineural HL, vestibular areflexia, and adolescent-onset retinitis pigmentosa (RP). In this study, we identified one case (Case 6) with compound heterozygous *MYO7A* variants who showed progressive HL and late-onset RP. His clinical phenotype of HL and RP was milder than that of typical USH1B cases. The novel variant *MYO7A*: c.1369G>A: p.A457T may cause a milder phenotype than that described in previous reports and cause a similar phenotype to that of Usher syndrome type III. The type classification in Usher syndrome is traditionally classified on the basis of clinical symptoms, not by the causative gene. Therefore, it is highly possible that new clinical phenotypes will emerge as information regarding the causative gene becomes clearer. This case is a good example of such phenotypic expansion.

The *ACTG1* gene is known as a genetic cause of autosomal dominant non‐syndromic HL (ADNSHL) (DFNA20/26). In previous reports, most cases of *ACTG1*-associated HL showed onset in the first or second decade, with the first group showing high-frequency HL progressing in all frequencies [21, 22]. In this study, the patient with *ACTG1*: c.833C>T:p.T278I variants became aware of her HL at age 30. Her hearing level thereafter gradually progressed to a profound level. Her son also carried the same variant and showed a similar phenotype. He received CI at the age of 40, and showed good performance after implantation.

The *EYA4* gene is known as a genetic cause of ADNSHL (DFNA10). The audiometric configuration for *EYA4*-associated HL was gradual high-frequency HL or flat-type HL [23, 24]. In this study, we identified two patients with different frame-shift variants. The age at onset of HL in these cases was in the 40 s, and the severity of HL was moderate to severe. The audiometric configuration types were flat and gently sloping, respectively, similar to those in previous reports.

The *COCH* gene is known to cause autosomal dominant and late-onset progressive sensorineural HL with vestibular dysfunction (DFNA9) [25, 26]. We identified four cases with *COCH* variants, and 3 of them carried the same variant (c.1115T>C:p. I372T). This variant has also been reported previously [27]. The same variant was reported from different Japanese ADNSHL families, and this variant may have spread from the same founder (founder mutation). Cochlin, the product of the *COCH* gene, has one LCCL domain and two vWFA domains. A mutation in the LCCL domain is reported to cause vertigo/dizziness more frequently than that in the vWFA domains [27]. The c.236A>G:p.H79R variant, which was identified as a novel variant in this study, is located in the LCCL domain. The phenotype of this case and his mother was late-onset progressive HL and dizziness, as previously reported.

The *MYO6* gene is known to be responsible for both ADNSHL (DFNA22) and ARNSHL (DFNB37) [28]. In a previous study, patients with *MYO6* variants showed late-onset mild-to-moderate progressive HL, and marked hearing deterioration occurred after the age of 40 [29]. In our study, the phenotypes of *MYO6*-associated HL varied as shown in Table 2.

The *KCNQ4* gene is known to be one of the most frequent causative genes for ADNSHL (DFNA2) [30]. It has been shown that DFNA2 results in high-frequency-involved HL. A progressive nature is a common feature among patients with *KCNQ4* mutations, regardless of the variant [31]. In this study, we identified 3 families with *KCNQ4* variants, with one of them being a novel frameshift variant (c.1656dupA:p.L553Tfs*11). The audiometric configuration of this patient showed sensorineural hearing impairment involving all frequencies.

As a notable result, we also identified one case of phenotypically mild Stickler syndrome with a homozygous *COL9A3* frameshift variant. The *COL9A3* gene, first reported as a genetic cause of multiple epiphyseal dysplasia, an autosomal dominant osteo-chondro-dysplasia, [32] was also shown to be responsible for Stickler syndrome in two previous reports [33, 34]. Faletra et al., reported an autosomal recessive Stickler syndrome family with three affected siblings, all of whom carried a homozygous frameshift variant in the *COL9A3* gene. All three individuals showed high-frequency HL and moderate-to-high myopia and amblyopia. Among the three affected siblings, the two elder brothers showed flat midface hypoplasia and a depressed nasal bridge, but the youngest sister in this family did not show these malformations. Hanson-Kahn et al., reported a patient with Stickler syndrome, who carried a homozygous *COL9A3* frameshift variant and showed moderate-to-severe sensorineural HL, severe myopia, and both tibial and femoral bowing at birth [34]. As *COL9A3* variants have been reported to cause non-syndromic HL [35], *COL9A3* variants may have a broad phenotypic range from mild non-syndromic HL to more severe syndromic phenotypes. Due to inter- and intra-familial phenotypic variability [34, 35], it is extremely difficult to perform diagnosis based on clinical phenotypes. The present case, the third reported to date, presenting only with HL, cataract and retinal detachment, can be classified as a very mild type of Stickler syndrome that could not be diagnosed without genetic diagnosis. At her first visit, she was diagnosed with non-syndromic HL as she had no noticeable symptoms. Later, however, as the *COL9A3* mutation was identified by genetic analysis, a detailed anamnestic re-evaluation of the associated symptoms of Stickler syndrome revealed a history of cataract and retinal detachment.

In conclusion, we performed genetic analysis of 48 Japanese patients with late-onset bilateral SNHL and identified the potential genetic causes of HL in 29 cases (60.4%). The present results clearly indicated that a significant portion of cases of late-onset bilateral SNHL may involve genetic causes, and the various causative genes can possibly be identified. From an etiological perspective, we could confirm the benefits of CI in cases in which the etiology is located within the cochlea, as shown in the *MYO7A*- and the *ACTG1*-associated cases. In addition, we could expand phenotypic descriptions as seen in (1) the *MYO7A*-associated case with a milder phenotype of Usher syndrome and (2) the phenotypically mild Stickler syndrome caused by a homozygous frameshift variant in the *COL9A3* gene. These two cases could not be diagnosed without genetic testing. Therefore, comprehensive genetic testing can be seen as a useful diagnostic tool for late-onset HL, and the accumulation of genetic findings will enable more accurate diagnosis and provide more appropriate treatment.
